# Repurposing of Doxycycline to Hinder the Viral Replication of SARS-CoV-2: From *in silico* to *in vitro* Validation

**DOI:** 10.3389/fmicb.2022.757418

**Published:** 2022-05-04

**Authors:** Rajaiah Alexpandi, Mathieu Gendrot, Gurusamy Abirami, Océane Delandre, Isabelle Fonta, Joel Mosnier, Richard Mariadasse, Jeyaraman Jeyakanthan, Shunmugiah Karutha Pandian, Bruno Pradines, Arumugam Veera Ravi

**Affiliations:** ^1^Laboratory in Microbiology and Marine Biotechnology, Department of Biotechnology, School of Biological Sciences, Alagappa University, Karaikudi, India; ^2^Parasitology and Entomology Unit, Department of Microbiology and Infectious Diseases, French Armed Forces Biomedical Research Institute, Marseille, France; ^3^IRD, SSA, AP-HM, VITROME, Aix-Marseille University, Marseille, France; ^4^IHU Méditerranée Infection, Marseille, France; ^5^National Reference Center for Malaria, Marseille, France; ^6^Structural Biology and Bio-Computing Laboratory, Department of Bioinformatics, Alagappa University, Karaikudi, India

**Keywords:** COVID-19, drug repurposing, doxycycline, SARS-CoV-2, *in silico*, *in vitro*

## Abstract

Since the rapid spread of coronavirus disease (COVID-19) became a global pandemic, healthcare ministries around the world have recommended specific control methods such as quarantining infected peoples, identifying infections, wearing mask, and practicing hand hygiene. Since no effective treatment for COVID-19 has yet been discovered, a variety of drugs approved by Food and Drug Administration (FDA) have been suggested for repurposing strategy. In the current study, we predicted that doxycycline could interact with the nucleotide triphosphate (NTP) entry channel, and is therefore expected to hinder the viral replication of SARS-CoV-2 RNA-dependent RNA-polymerase (RdRp) through docking analysis. Further, the molecular dynamics results revealed that the RdRp-Doxycycline complex was structurally relatively stable during the dynamic period (100 ns), and its complex maintained close contact with their active catalytic domains of SARS-CoV-2 RdRp. The molecular mechanics Poisson–Boltzmann surface area (MM-PBSA) calculation of binding free energy also showed that the doxycycline has worthy affinities with SARS-CoV-2 RdRp. As expected, doxycycline effectively inhibited the viral replication of IHU strains of SARS-CoV-2 (IHUMI-3 and IHUMI-6), identified from the hospitalized patients in IHU Méditerranée Infection (IHUMI), Marseille, France. Moreover, doxycycline inhibited the viral load *in vitro* at both on-entry and after viral entry of IHU variants of SARS-CoV-2. The results suggest that doxycycline exhibits strains-dependant antiviral activity against COVID-19. As a result, the current study concludes that doxycycline may be more effective in combination with other drugs for better COVID-19 treatment efficacy.

## Introduction

Coronavirus disease 2019 (COVID-19) is a respiratory illness associated with signs such as a dry cough, fever, shortness of breath, muscle aches, fatigue, and dyspnea, leading to pneumonia (Wu et al., 2020). Acute respiratory distress syndromes (ARDS) such as hypercytokinemia, severe acute respiratory syndrome, lymphopenia, disseminated intravascular coagulation, renal failure, and mortality can occur in severe condition (Gostic et al., 2020). In severe situations, lung inflammation becomes so severe that it produces septic shock, which results in mortality owing to a decrease in blood pressure and oxygen deprivation. The pathognomonic symptoms as well as severity of COVID-19 vary from patient to patient (Menni et al., 2020; Shah et al., 2020). The current outbreak of COVID-19 was declared a global emergency of international concern by the WHO due to its rapid spread, with a measured basic reproductive number (R_*o*_) of 2.2 (Rafiq et al., 2020). As of 10 August 2021, the WHO reports that 203,295,170 cases have been definitively diagnosed around the world, including 4,303,515 deaths.

COVID-18 has no specific treatment available at this time. Because COVID-19 has the potential to be a devastating disease for the entire civilization, healthcare authorities around the world have recommended a number of preventative measures, such as quarantining infected patients, wearing proper masks, regular hand sanitization on a regular basis, and rapid testing and diagnosis of those suspected of being infected (Li et al., 2020). Simultaneously, scientists are examining COVID-19 vaccines and therapeutic treatments (Pawar, 2020). However, novel medicines on the other hands require months, if not years, to reach the commercial market (Abd El-Aziz and Stockand, 2020). As a result, researchers are now developing a repurposing strategy to identify appropriate therapeutics among currently available drugs, to be used against COVID-19 (Phadke and Saunik, 2020).

Severe acute respiratory syndrome coronavirus 2 (SARS-CoV-2) is a positive-sense enveloped and single-strand RNA virus, which belongs to the *Coronaviridae* family and *Betacoronavirus* genus (Andreani et al., 2020). The novel SARS-CoV-2 is highly similar to SARS-CoV and MERS, and the genome size is approximately 30 kb (Zhang and Holmes, 2020; Zou et al., 2020). Among the SARS-CoV-2 viral proteins, the 3-chymotrypsin like protease (3CLpro) is an imperative enzyme that plays a fundamental role in the dispensation of viral polyproteins, which are necessary for viral development and their pathogenesis. Impeding 3CLpro activity could hinder the viral replication of SARS-CoV-2 and has been seen as a promising strategy for inventing suitable therapeutic agents against COVID-19 (Rathnayake et al., 2020). As a result, Owen et al. (2021) have recently reported that PF-07321332, an orally accessible SARS-CoV-2 main protease inhibitor, showed excellent antiviral activity with *in vivo* safety outline against COVID-19. Similarly, RNA-dependent RNA-polymerase (RdRp), a decisive polymerase enzyme that is crucial in the replication process, has been seen as a promising drug target for discovering potent antiviral agents against COVID-19 (Gao et al., 2020).

**FIGURE 1:**
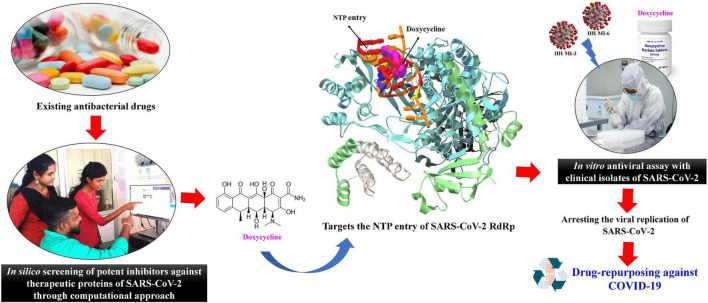
Graphical representation of the present study for the drug-repurposing of doxycycline against COVID-19.

Due to the lack of a COVID-19 therapy, a variety of existing drugs are being used to treat the symptoms of patients with COVID-19 in the form of a drug-repurposing approach. Many more drugs, like antimicrobial, are now undergoing human trials to determine their efficacy against COVID-19 (Cimolai, 2020). Andreani et al. (2020) have reported that the antibacterial agent azithromycin showed synergistic anti-SARS-CoV-2 activity combined with hydroxychloroquine. Furthermore, Gautret et al. (2020) also demonstrated that the results of the combination of hydroxychloroquine and azithromycin in human trials were positive; all patients taking the combination were virologically restored to health within 6 days of treatment. In this study, we identified doxycycline as a potent antiviral compounds for SARS-CoV-2 through *in silico* and *in vitro* approach (Figure 1).

## Materials and Methods

### Ligand Preparation

Drug profile of the existing antibacterial drugs (321 drugs) was obtained from the DrugBank server^1^. The canonical SMILES of the antibacterial drugs were obtained from the PubChem server. Then, the PDB format three-dimensional (3D) structure of the antibacterial drugs was generated using the Online SMILES Translator server (Alexpandi et al., 2019).

### Protein Preparation

The 3D structure of SARS-CoV-2 RdRp-Nsp7-Nsp8 complex (PDB ID: 7AAP) was downloaded from the RCSB PDB database. The homology-modeled protein of SARS-CoV-2 RdRp was obtained from Prof. Hua Li, HUST, China (Wu et al., 2020). Energy minimization was performed using YASARA software and protein preparation was performed using the AutoDock Tools Version 1.5.6 (Balasubramaniam et al., 2019). The natural ligands present in the protein structures were isolated by deleting atomic orchestrates of the PDB file. During protein preparation, all the water molecules were deleted, and hydrogen polarities and Kollman charges were allocated, using AutoDock Tools Version 1.5.6 platform. Protein structures were then translated from PDB file to PDBQT file format to perform the docking analysis with AutoDock vina.

### Molecular Docking

First, the top binding compounds with targeted proteins were scrutinized by virtual screening using the iGEMDOCK tool. After the finding of best compounds, the binding mode of top-scoring compounds with the active site of SARS-CoV-2 RdRp protein was studied using AutoDock Vina (Alexpandi et al., 2020). Ligands were independently docked with the therapeutic proteins adopting a flexible mode under rigid conditions. The grid size was set at 126 × 126 × 126 (x, y, and z) points with a grid spacing 1 Å, and the grid center was set based on the proteins. The docking was performed with AutoDock vina and prepared file was saved in PDBQT format. The binding scores of ligands were obtained in the form of Kcal/mol calculated using the Autodock vina scoring algorithm. The output of the docking files was then saved in PDB format for post-docking analysis. Post-docking analysis, including monitoring interacted amino acid residues, was performed using Maestro (Schrödinger) software. The predicted drugs in the present study were then compared with reported anti-COVID-19 agents such as lopinavir and remdesivir triphosphate (Cheng et al., 2020).

### Molecular Dynamic Simulation Studies

To confirm the stability of the ligand-bound complexes of therapeutic targets of SARS-CoV-2, molecular dynamic simulation studies were performed for 100 ns using Gromacs software. The ligand topologies were obtained from the PRODRUG server. All ligand-bound structures were solvated with SPC216 water molecules and periodic boundary conditions were applied to all directions of cubic boxes with 1.0 and 1.2 nm dimensions. To obtain the precise electrostatic values during the dynamics, all of the systems (net charge) were neutralized with appropriate Na^+^ ions. Steepest Descent Algorithm (SDA) and Conjugate Gradient Algorithm (CGA) were used to perform energy minimization (50,000 steps) with a tolerance of 1,000 kJ mol^–1^ nm^–1^, followed by a two-step equilibrium phase (NPT-particles, volume and temperature; NVT-particles, pressure and temperature combinations). Bond constraints of the molecular simulations were maintained by the LINear Constraint Solver (LINCS) algorithm. The Particle Mesh Eshwald (PME) method was used to compute the long-range Coulomb interactions with the default cut-off parameter of 0.12 nm. Temperature (310 K) was regulated using the V-rescale weak coupling method and the Parrinello–Rahman (PR) method was used to equilibrate [pressure (1 atm), temperature, density, and total energy] the systems. All the pre-equilibrated systems were subjected to molecular dynamic simulation for 100 ns and the coordinates were saved every 2 ps (Choubey et al., 2020).

### MM-PBSA Calculation for Protein-Ligand Complexes

In order to calculate the binding-free energies (affinities) of the complexes during the dynamics, MM-PBSA calculation was performed. Here, the trajectories obtained from last 10 ns were considered to calculate Gibbs free energy using g_mmpbsa tool (combined in the GROMACS software) and the following equation (Mariadasse et al., 2020).


$$\Delta \,{\text{G}}_{\text{bind}}={\text{G}}_{\text{complex}}-\left({\text{G}}_{\text{protein}}+{\text{G}}_{\text{ligand}}\right)$$

Where, ΔG_complex,_ is defined as total energy, ΔG_protein_ and ΔG_ligand_ are defined to reach the total binding-free energy of the protein and ligand.

### *In vitro* Confirmation of Anti-SARS-CoV-2 Activity

#### Drugs, the Virus and Cells

Stock solutions of doxycycline hyclate (Sigma Aldrich, St Quentin Fallavier, France) were prepared in methanol and diluted in Minimum Essential Media (MEM, Gibco, ThermoFischer, Waltham, MA, United States), resulting in seven final concentrations ranging from 0.1 to 100 μM. Chloroquine diphosphate (Sigma Aldrich) and remdesivir (Apollo Scientific, Manchester, United Kingdom) were used as positive anti-SARS-CoV-2 compounds. Stock solutions of chloroquine were prepared in water and remdesivir in DMSO/water 10%. In total two clinically-isolated SARS-CoV-2 strains (IHUMI-3 and IHUMI-6), collected in hospitalized patients during the first COVID-19 outbreak in March 2020 in IHU Méditerranée Infection (IHUMI), Marseille, France, were maintained in production in Vero E6 cells (American type culture collection ATCC CRL-1586) in MEM with 4% fetal bovine serum and 1% glutamine (complete medium) (Gautret et al., 2020). All the experiments with SARS-CoV-2 were performed in the Biosafety Level (BSL)-3 Laboratory.

#### *In vitro* Antiviral Assay

Briefly, 96-well plates were prepared with 10^4^ cells/mL of Vero E6 (200 μL per well), as previously described (Andreani et al., 2020). Doxycycline concentrations were added 4 h before infection. Vero E6 cells were infected with IHUMI-3 or IHUMI-6 strains at an MOI (multiplicity of infection) of 0.8. Reverse transcription polymerase chain reaction (RT-PCR) was used 48 h post-infection to estimate the replication using the Superscript III platinum one step with the Rox kit (Invitrogene) after extraction with the BIoExtract SuperBall kit (Biosellal, Dardilly, France). In the RT-PCR system for SARS-CoV-2 detection, the envelope protein (E)-encoding gene was used. The following primers and probe were used: E_forward (ACAGGTACGTTAATAGTTAATAGCGT), E_probe (FAM-ACACTAGCCATCCTTACTGCGCTTCG-TAMRA), E_reverse (ATATTGCAGCAGTACGCACACA) (Corman et al., 2020). The percentage of inhibition of SARS-CoV-2 replication was estimated for each drug concentration as follows:

(mean CT_drug concentration_ − mean CT_control 0%_)/(mean CT_control 100%_ − mean CT_control 0%_) × 100.

EC_50_ (median effective concentration) and EC_90_ (90% effective concentration) were calculated with the inhibitory sigmoid Emax model, which estimated the EC_50_ and EC_90_ through non-linear regression by using a standard function of the R software (ICEstimator version 1.2). The EC_50_ and EC_90_ values resulted in a mean of 7–10 independent experiments.

#### Determination of the Inhibition Stage

Furthermore, to identify the inhibition stage of doxycycline on the entry and post-entry of SARS-CoV-2 was evaluated with IHUMI-3 strain at a concentration of 10 μM. For the “full-time treatment,” Vero E6 cells were infected with the IHUMI-3 strain for 48 h after pre-incubation of the cells with the tested drugs for 4 h. For the “entry” treatment, the cells were infected for 2 h after pre-incubation for 4 h and then the virus-drug mixture was replaced with fresh medium maintained for 46 h. For the “post-entry” treatment, the cells were infected for 2 h and then incubated with drug for 46 h. The percentage of inhibition of SARS-CoV-2 replication by 10 μM of drug was estimated for each drug concentration as following:

(mean CT_drug concentration_ − mean CT_control 0%_)/(mean CT_control 100%_ − mean CT_control 0%_) × 100.

## Results

### Screening of Potent Inhibitors for SARS-CoV-2 RdRp

**FIGURE 2:**
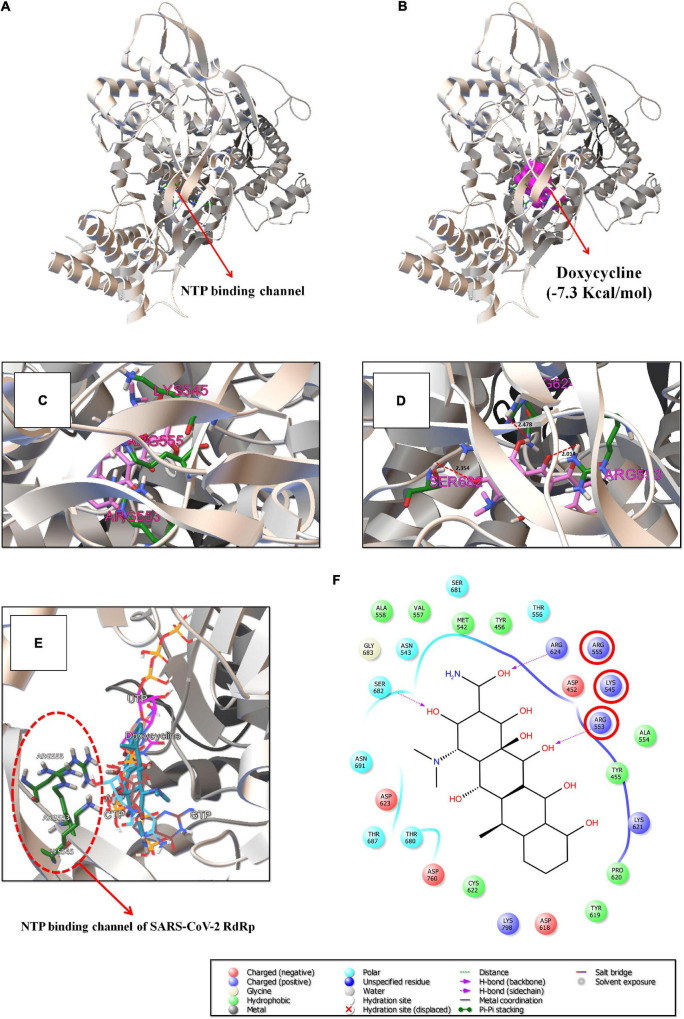
Panel **(A)** shows the position of the NTP binding channel of SARS-CoV-2 (set of hydrophilic residues such as Lys545, Arg553, and Arg555). Panels **(B,C)** indicate the overview and close-up view of the binding interaction of doxycycline (–7.3 Kcal/mol) with the NTP binding channel of SARS-CoV-2. Panel **(D)** indicates the hydrogen bond formation of doxycycline with Arg553, Arg624, and Ser682 residues of SARS-CoV-2 RdRp at 2.014 Å, 2.478 Å, and 2.354 Å distances. Panel **(E)** indicates the antagonistic action of doxycycline on the binding sites of the parental-nucleotides of SARS-CoV-2 RdRp. Panel **(F)** demonstrates the interacted amino acid residues of SARS-CoV-2 RdRp with doxycycline.

**FIGURE 3:**
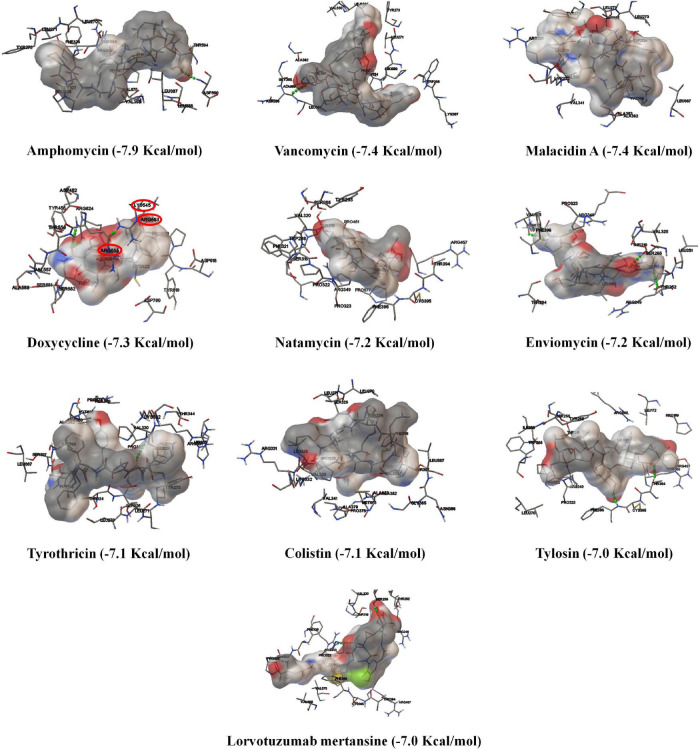
Schematic representation of mode of action of doxycycline against SARS-CoV-2. The binding patterns and aminoacid interactions of the best scoring antibacterial drugs with SARS-CoV-2 RdRp. The red circle-marking indicates the interaction with the active site (NTP binding channel) of SARS-CoV-2 RdRp.

In the finding of SARS-CoV-2 RdRp-inhibitors, the existing antibacterial drugs such as amphomycin, vancomycin, malacidin A, doxycycline, natamycin, enviomycin, tyrothricin, colistin, tylosin, and lorvotuzumab mertansine were identified as the top binding drugs for RdRp (Figure 2 and Supplementary Table 1). Among the top-10 compounds, only the antibacterial drug doxycycline (−7.3 Kcal/mol) could inhibit the activity of SARS-CoV-2 RdRp by interacting with the NTP entry channel (Figure 3), as like remdesivir triphosphate (−7.8 Kcal/mol) (Supplementary Figure 1). The NTP entry channel is composed of hydrophilic amino acids, including Lys545, Arg553, and Arg555 (Figure 3). The results reveal that doxycycline has a similar binding site at SARS-CoV-2, similar to parental nucleotides with almost similar binding energy (Figure 3).

As shown in Figure 3, doxycycline strongly builds nine hydrophobic bonds (Ala558, Val557, Met542, Tyr456, Ala554, Tyr455, Pro620, Tyr619, and Cys622), seven polar bonds (Asn691, Thr687, Thr680, Ser682, Asn543, Ser681, and Thr556), four negative-charged bonds (Asp623, Asp760, Asp618, and Asp452), and six unspecified bonds with Arg553, Lys545, Arg555, Lys621, Lys798, and Arg624. Importantly, doxycycline creates three hydrogen bond interactions with Arg553, Arg624, and Ser682 residues at 2.014 Å, 2.478 Å, and 2.354 Å distance, respectively (Figure 3). Based on the obtained results, we anticipate that the existing antibacterial drug, doxycycline, will interrupt the NTP entry channel and arrest viral replication, as is the case with remdesivir triphosphate, a known anti-COVID-19 agent (Eastman et al., 2020).

### Molecular Dynamics Studies of Structural Stability

**FIGURE 4:**
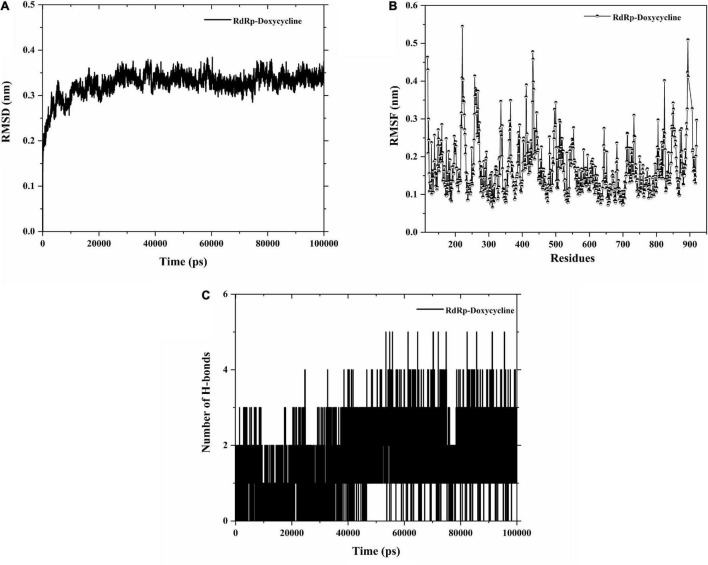
Panels **(A,B)** show the RMSD and RMSF profiling of the RdRp-Doxycycline complex during 100 ns simulation. Panel **(C)** indicates the hydrogen bond routes of the RdRp-Doxycycline complex during 100 ns simulation.

The structural stability of RdRp-Doxycycline complex was analyzed using root mean square deviation (RMSD), root mean square fluctuation (RMSF), and hydrogen bond (H-bonds) interactions. The RMSD and RMSF plots of the RdRp-Doxycycline complex revealed deviation and residual fluctuation values in the range of 0.25–0.35 nm and 0.1–0.55 nm, respectively, suggesting the RdRp-Doxycycline complex was very stable during the dynamic periods. The RMSD profile reveals that the structural behavior of RdRp-Doxycycline maintains their structural integrity during the 100 ns simulation (Figure 4A). The RMSF profile also revealed the structural stability of the RdRp-Doxycycline without major structural deviation (Figure 4B). Furthermore, the H-bonds plots showed that the complex has five H-bond interactions in the active site pocket (Figure 4C). These H-bonds were found to constantly interact with catalytic residues such as Arg553, Asp761, Asp760, Lys621, and Arg624, suggesting that doxycycline could inhibit the function of SARS-CoV-2 RdRp (Table 1).

**TABLE 1:** Potential H-bond interactions found in the proposed complexes.

Complex	H-bond interactions
RdRp-Doxycycline	Arg553, Arg555, Lys545, Asp623, Ser682, Thr556, Ala554, Arg624, Thr556, Lys621, Glu167, Asp618, Ala688, Asp760, and Asp761.

### MM-PBSA Binding Free Energy Calculation

The binding-free energy calculation also helps to understand the stability of protein-ligand complexes in terms of affinity during molecular dynamics. It calculates major interaction energies such as electrostatic, Van der Waals, solvent accessible surface area, and total energy of the protein-ligand complexes. In Table 2, the RdRp-Doxycycline complex showed higher electrostatic interaction energy (−216.693 kj/mol), Van der Waals energy (−469.363 kj/mol), SASA energy (−17.681 kj/mol), and total binding free energy (−163.980 kj/mol). Significantly, Van der Waals energy is more favorable in the RdRp-Doxycycline complex due to the desolvation effect of active site residues in the cavity, while the electrostatic interaction energy showed relatively lesser affinity due to the attractive and repulsive parts of the complex. These two interaction energies are important to estimate the stable conformation of the RdRp-Doxycycline complex. Similarly, solvent accessible surface area (SASA) calculates the cavitations, dispersion, and repulsion energies of the RdRp-Doxycycline complex suggesting that the complex is stable during the dynamics. Hence, the hydrophobic, polar and negative charged interactions are so much important for RdRp-drug binding. Overall, the obtained results reveal that doxycycline has a higher binding affinity toward the complex formation with the therapeutic protein of SARS-CoV-2.

**TABLE 2:** MM-PBSA binding free energy calculation for the formed complexes.

Complexes	Electrostatic kJ/mol	Van der Waals kJ/mol	SASA Energy kJ/mol	Total Energy kJ/mol
RdRp-Doxycycline	−216.693	−469.363	−17.681	−163.980

### *In vitro* Antiviral Activity of Doxycycline Against SARS-CoV-2

**FIGURE 5:**
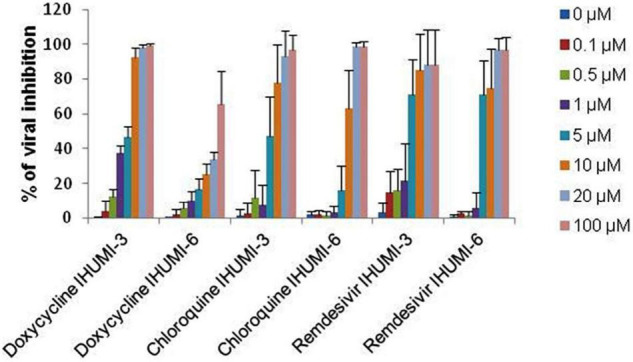
Anti-SARS-CoV-2 activity of doxycycline, chloroquine and remdesivir in % of antiviral inhibition on IHUMI-3 and IHUMI-6 clinically-isolated strains (error bars represent standard deviation of 7–10 independent experiments).

The antiviral activity was evaluated using an *in vitro* experiment with the clinical isolates (IHUMI-3 and IHUMI-6) of SARS-CoV-2. The results revealed that the tested antibacterial drug (doxycycline) showed efficient antiviral activity against the clinical isolates (IHUMI-3 and IHUMI-6) of SARS-CoV-2 at lower concentration (Table 3). The results showed that the dose-dependent responses were observed for doxycycline, chloroquine, and remdesivir against SARS-CoV-2, as shown in Figure 5. The median effective concentration (EC_50_) and 90% effective concentration (EC_90_) of doxycycline against the IHUMI-3 strain are 5.8 ± 1.6 μM and 21.7 ± 5.9 μM, respectively, at 0.8 MOI. Consistently, the EC_50_ and EC_90_ of doxycycline against IHUMI-6 are 35.4 ± 12.4 μM and 361 ± 69 μM at 0.8 MOI. The results obtained reveal that the antiviral behavior of doxycycline against SARS-CoV-2 was strain-dependent. Overall, the *in vitro* data strongly validated the *in silico* result and proved the antiviral activity of doxycycline against SARS-CoV-2. Doxycycline (35.4 μM) was much less effective than remdesivir (4.3 μM) and chloroquine (8.7 μM) against IUHMI-6. There was no difference between *in vitro* responses to doxycycline, remdesivir, or chloroquine against IHUMI-3 (*p* = 0.129, Kruskal–Wallis test). Simultaneously, other top binding-compounds, such as amphomycin, vancomycin, malacidin A, natamycin, enviomycin, tyrothricin, colistin, tylosin, and lorvotuzumab mertansine were found to be ineffective against SARS-CoV-2.

**TABLE 3:** *In vitro* antiviral activity of tested antibacterial drugs against the clinical isolates of SARS-CoV-2.

Drug name	Median effective concentration (EC)	Clinical isolates of SARS-CoV-2	
		IHUMI-3	IHUMI-6
Doxycycline	EC_50_ in μM ± SD	5.8 ± 1.6 μM	35.4 ± 12.4 μM
	EC_90_ in μM ± SD	21.7 ± 5.9 μM	361 ± 69 μM
Remdesivir (Positive control)	EC_50_ in μM ± SD	2.7 ± 1.5 μM	4.3 ± 1.6 μM
	EC_90_ in μM ± SD	7.7 ± 2.7 μM	15.0 ± 6.1 μM
Chloroquine (Positive control)	EC_50_ in μM ± SD	5.2 ± 3.3 μM	8.7 ± 2.7 μM
	EC_90_ in μM ± SD	11.3 ± 8.2 μM	13.7 ± 5.4 μM

**FIGURE 6:**
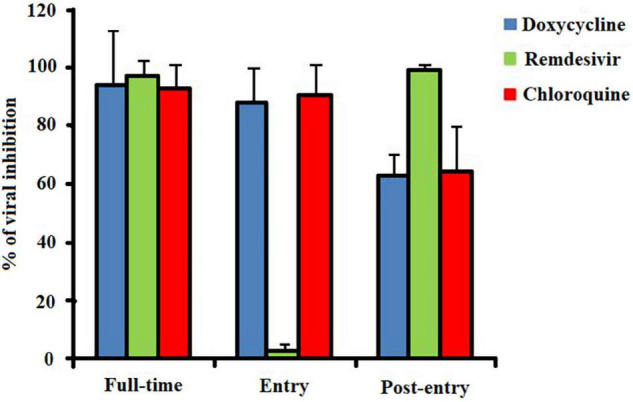
*In vitro* antiviral activity of doxycycline, chloroquine, and remdesivir at 10 μM against the SARS-CoV-2 IHUMI-3 strain. For the “full-time” treatment, Vero E6 cells were infected with the IHUMI-3 strain for 48 h after pre-incubation of the cells with one of the three drugs for 4 h. For the “entry” treatment, the cells were infected for 2 h after pre-incubation for 4 h and then the virus-drug mixture was replaced with fresh medium maintained for 46 h. For the “post-entry” treatment, the cells were infected for 2 h and then incubated with drug for 46 h.

Furthermore, the effects of doxycycline, chloroquine, or remdesivir on entry and post-entry of SARS-CoV-2 was evaluated using IHUMI-3 strain at a concentration of 10 μM. The result shows that doxycycline well interacted at both entry and post-entry stages of SARS-CoV-2 infection in Vero E6 cells, as chloroquine did (Figure 6). In reverse, remdesivir, which is an anti-SARS-CoV-2 drug, interacted only at post-entry stage. Hence, the *in vitro* results strongly suggest that the doxycycline could be a potent antiviral compound for the development of therapeutic medication against COVID-19 as like chloroquine and remdesivir.

## Discussion

Several antiviral agents have been assessed against viral proteases, polymerases, and entry proteins of SARS-CoV-2 for treating COVID-19 (Rosa and Santos, 2020). The described anti-COVID-19 drugs are largely inhibitors for 3CLpro or RdRp. For instance, in remdesivir, a prodrug targets budding viral-RNA by blocking the activity of SARS-CoV-2 RdRp, which leads to the arrest of RNA synthesis (Elfiky, 2020). Likewise, favipiravir and ribavirin have also demonstrated their potential against COVID-19 (Cai et al., 2020). Owing to the crucial role of viral proteins, SARS-CoV-2 3CLpro and RdRp have been seen as a hopeful therapeutic approach to treating COVID-19 (Ahmed et al., 2020; Wu et al., 2020).

During the development of drugs against COVID-19, SARS-CoV-2 RdRp has been considered as an important therapeutic target owing to the central role it plays in RNA synthesis from the RNA-template during replication and transcription (Elfiky, 2020). Therefore, SARS-CoV-2 RdRp is a key when it comes to nucleotide analog-drugs such as remdesivir, exhibits positive results for drug-repurposing treatment against COVID-19 (Choy et al., 2020). Further, nucleotide analog-based antiviral drugs were unable to demonstrate significant reverse effects on humans (Dong et al., 2020). The RdRp-complex is activated through polymerase motifs A to G, located in the 549–776 regions, which relate to RNA-mediated 5′–3′ polymerase activity. Similarly to other RdRp, the NTP entry channel and budding strand assemble at a central hollow where RdRp-motifs mediate RNA-template based replication by the NTP binding site located at Lys545, Arg553, and Arg555 (Gao et al., 2020). The nucleotide analogs, namely favipiravir and remdesivir, arrest NTP binding, and thereby showed their antiviral activity against COVID-19 (Gordon et al., 2020).

In the current study, the *in silico* results obtained reveal that doxycycline has the potential to interrupt with the NTP entry channel of SARS-CoV-2 RdRp (Figure 3). Furthermore, the binding site of doxycycline with the NTP entry channel of RdRp was confirmed by re-docking with the SARS-CoV-2 RdRp-Nsp7-Nsp8 complex (PDB ID: 7AAP). The result also showed that doxycycline was able to interfere with NTP entry with low-binding energy (−10.5 Kcal/mol), and hence we believed to arrest the viral replication of SARS-CoV-2. In molecular simulation analysis, the RMSD and RMSF graphs illustrated no major fluctuation for the RdRp-Doxycycline complex (Figure 4). The structural stability of the complexes did not exceed 0.5 nm, suggesting that all the complexes were architecturally highly stable, indicating that the doxycycline could be active against SARS-CoV-2. Furthermore, the MM-PBSA calculation revealed that the RdRp-Doxycycline complex possesses virtuous affinities such as high-electrostatic interaction, Van der Waals interaction, and SASA energy toward to the catalytic domains of SARS-CoV-2 RdRp, leading to greater attention being paid to assessing the potential of doxycycline against SARS-CoV-2.

As expected, the *in vitro* experiment confirmed the antiviral activity of doxycycline against SARS-CoV-2 at low micromolar concentrations (Table 3). The obtained results revealed that doxycycline may have the potential to hinder the viral replication of both the IHUMI-3 and IHUMI-6 strains of SARS-CoV-2. The EC_50_ and EC_90_ values of doxycycline were found to be 5.8 ± 1.6 μM and 21.7 ± 5.9 μM against the IHUMI-3 strain at an MOI of 0.8, in coherence with previous data (Gendrot et al., 2020). Doxycycline is as effective *in vitro* as remdesivir or chloroquine against IHUMI-3 strain (*p* = 0.129, Kruskal–Wallis test). However, doxycycline is less significantly active against the IHUMI-6 strain of SARS-CoV-2 (EC_50_ = 35.4 ± 12.4 μM and EC_90_ = 361 ± 69). Moreover, the doxycycline inhibited the viral load *in vitro* at both on entry and after viral entry of SARS-CoV-2 (Figure 6). Based on *in silico* and *in vitro* experiments, the current study suggests that doxycycline could be a feasible drug candidate for a potential COVID-19 treatment.

The lack of *in vivo* study or human trial to address the therapeutic action of doxycycline against COVID-19 is an impeding limitation of this study. As a part of drug repurposing actions, doxycycline was known to have *in vitro* activity against SARS-CoV-2 (Gendrot et al., 2020). The possible therapeutic action of doxycycline is due to the pleiotropic effects against the general pathways involved in viral infection, replication, and associated over expression of the zinc finger antiviral protein, which binds to viral messenger RNAs and inhibits translation of viral RNAs (Malek et al., 2020). Doxycycline has anti-inflammatory, antibacterial, and possibly antiviral effects, and so was believed as potential treatments for COVID-19. Malek and Granwehr (2021) have reported doxycyline as an alternative to azithromycin in elderly patients for the treatment of COVID-19. In the majority of cases, early dose of doxycycline treatment was related with improved clinical outcomes, decreased hospitalizations, and decreased mortality. However, larger randomized control trials are needed to evaluate the outcome of doxycycline treatment in moderate to severe COVID-19 infections (Alam et al., 2020). Yates et al. (2020) also reported that the doxycycline resolved the symptoms of high-risk COVID-19-positive patients with comorbid pulmonary disease. Unfortunately, the randomized evaluation of COVID-19 therapy trial recently reported that the use of doxycycline treatment in patients hospitalized with severe COVID-19 condition had no significant clinical assistance (Butler et al., 2021). It may be due to the development of resistance as well as the emergence of the variants among SARS-CoV-2 strains. Our results also revealed that the *in vitro* antiviral potential of doxycycline was differed based on the strains of SARS-CoV-2. On the other hand, the patients with COVID-19 infection who received doxycycline with ivermectin recovered faster than those who received placebo, were less likely to progress to severe illness, and were much more likely to test negative for COVID-19 (Mahmud et al., 2021). Overall, the pilot study suggests that doxycycline might be valuable while combination with other drugs for the treatment of COVID-19.

## Conclusion

Owing to its potential ability to target the therapeutic protein of SARS-CoV-2 *in silico*, doxycycline was predicted as the potent compound to arrest the viral replication through docking analysis. Molecular dynamics studies also revealed that the RdRp-doxycycline complex is architecturally highly stable and its complexes maintain close contact with their active catalytic domains. The MM-PBSA calculation also recommends that doxycycline has well-intentioned binding affinities with the catalytic domains of RdRp, suggesting that the doxycycline could be the potent antiviral compound against SARS-CoV-2. As predicted, doxycycline has the potential to hinder the viral replication of SARS-CoV-2 at low concentration. The *in vitro* results clearly demonstrate that doxycycline effectively arrested the viral replication of both IHUMI-3 and IHUMI-6 strains SARS-CoV-2 at low concentrations. Based on the results, the present study suggests that doxycycline could be very useful in combination with other anti-SARS-CoV-2 drugs for the treatment of COVID-19. However, further *in vivo* studies with animal models, followed by human trials, are required to authenticate the clinical effectiveness of doxycycline against COVID-19.

## Data Availability Statement

The original contributions presented in the study are included in the article/Supplementary Material, further inquiries can be directed to the corresponding author/s.

## Author Contributions

RA contributed to conceptualization, performed the *in silico* experiments, data analysis, and writing-original draft. GA performed the *in silico* experiments. MG, OD, IF, and JM performed the *in vitro* experiments with SARS-CoV-2. RM contributed in molecular simulation. JJ contributed in reviewing-original draft and data analysis. SP contributed in reviewing-original draft. BP and AR contributed in supervision, data analysis, and reviewing-original draft. All authors contributed to the article and approved the submitted version.

## Conflict of Interest

The authors declare that the research was conducted in the absence of any commercial or financial relationships that could be construed as a potential conflict of interest.

## Publisher’s Note

All claims expressed in this article are solely those of the authors and do not necessarily represent those of their affiliated organizations, or those of the publisher, the editors and the reviewers. Any product that may be evaluated in this article, or claim that may be made by its manufacturer, is not guaranteed or endorsed by the publisher.

## Footnotes

1
https://www.drugbank.ca/categories/DBCAT000104
