# Supplementary material for: Repurposing of Doxycycline to Hinder the Viral Replication of SARS-CoV-2: From in silico to in vitro Validation
Source: Front Microbiol. 2022 May 4;13:757418. doi: 10.3389/fmicb.2022.757418 (PMC9115549; doi:10.3389/fmicb.2022.757418)
Supplement: Supplementary file 1 [file Data_Sheet_1.PDF]

Supplementary Material

Repurposing of doxycycline to hinder the viral replication of SARS-CoV-2: From *in silico* to *in vitro* validation

Rajaiah Alexpandi[1], Gurusamy Abirami[1], Mathieu Gendrot[2,3,4], Océane Delandre[2,3,4], Isabelle Fonta[2,3,4,5], Joel Mosnier[2,3,4,5], Richard Mariadasse[6], Jeyaraman Jeyakanthan[6], Shunmugiah Karutha Pandian[1], Bruno Pradines[2,3,4,5]*, and Arumugam Veera Ravi[1]*

[1]Lab in Microbiology and Marine Biotechnology, Department of Biotechnology, School of Biological Sciences, Science Campus, Alagappa University, Karaikudi-630 003, India.

[2]Parasitology and Entomology Unit, Department of Microbiology and Infectious Diseases, French Armed Forces Biomedical Research Institute, Marseille, France.

[3]Aix Marseille Univ, IRD, SSA, AP-HM, VITROME, Marseille, France.

[4]IHU Méditerranée Infection, Marseille, France.

[5]National Reference Center for Malaria, Marseille, France.

[6]Structural Biology and Bio-Computing Lab, Department of Bioinformatics, Alagappa University, Karaikudi- 630 003, Tamil Nadu, India.

*** Corresponding authors:**

aveeraravi@rediffmail.com (A.V.R.); bruno.pradines@gmail.com (B.P.)

Supplementary Figures

Figure S1 (a) & (b) shows the binding region and close-up view of the interacting amino acids of the reported anti-COVID-19 drug (triphosphate form of remdesivir) (-7.8 Kcal/mol) with SARS-CoV-2 RdRp. (c) visualizes the interaction with the NTP entry channel of SARS-CoV-2 RdRp (a set of hydrophilic residues such as Lys545, Arg553, and Arg555) by remdesivir. (d) Hydrogen bond formation of remdesivir triphosphate with Lys551, Arg553, Lys621, and Lys798 residues of RdRp at 2.354, 2.502, 2.072, and 2.011 Å distance, respectively. (e) Illustrates the interacted aminoacid residues of SARS-CoV-2 RdRp with remdesivir triphosphate.

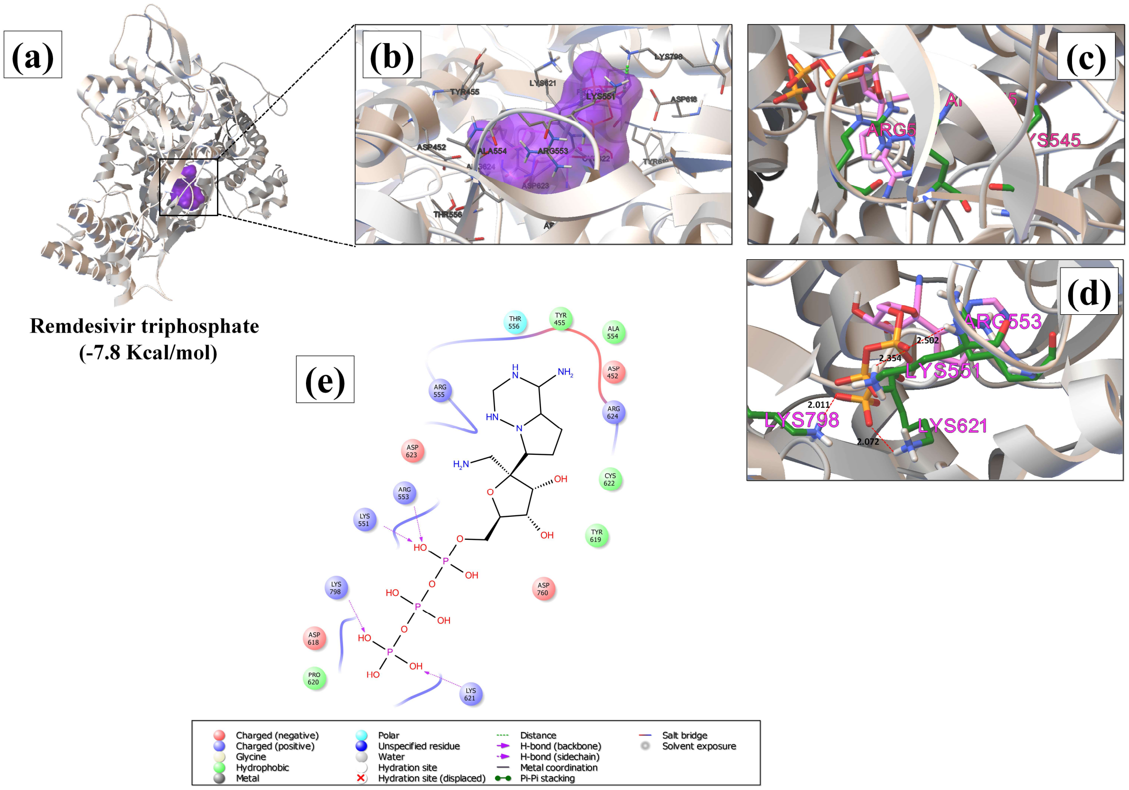

Supplementary Table

Table S2 iGEMDOCK-based virtual-screening of SARS-CoV-2 RdRp inhibitors from the antibacterial-drugs available in the DrugBank database.

PubChem ID	DrugBank Accession Number	Drug Name	Binding Energy (Kcal/mol)
**91663250**	**DB11499**	**Amphomycin**	**-133.6**
**14969**	**DB00512**	**Vancomycin**	**-129.5**
**132274667**	**DB14051**	**Malacidin A**	**-129.3**
**54671203**	**DB00254**	**Doxycycline**	**-128.7**
**5284447**	**DB00826**	**Natamycin**	**-126.4**
**3000546**	**DB08993**	**Enviomycin**	**-125.3**
**452550**	**DB13503**	**Tyrothricin**	**-124.9**
**44144393**	**DB00803**	**Colistin**	**-124.2**
**5280440**	**DB11475**	**Tylosin**	**-122.5**
**139033611**	**DB12089**	**Lorvotuzumab mertansine**	**-121.7**
54686187	DB13264	Penimepicycline	-121.2
6323289	DB01764	Dalfopristin	-120.8
3081545	DB04626	Apramycin	-120.3
5282242	DB01415	Ceftibuten	-119.9
54707177	DB00256	Lymecycline	-119.8
5479530	DB01212	Ceftriaxone	-118.3
9852981	DB06590	Ceftaroline fosamil	-117.2
6324616	DB11753	Rifamycin	-116.9
54686904	DB00560	Tigecycline	-116.9
5280965	DB00681	Amphotericin B	-116.5
65755	DB13821	Ceftezole	-116.2
54675785	DB00931	Metacycline	-116
56842231	DB13633	Mepartricin	-115.9
6450531	DB11405	Eprinomectin	-115.3
16186054	DB02975	GE-2270A	-115
135483770	DB00314	Capreomycin	-114.7
2639	DB13667	Cefozopran	-114.6
6915944	DB00535	Cefdinir	-114.2
439369	DB11512	Dihydrostreptomycin	-114
135398735	DB01045	Rifampicin	-113.5
9832750	DB11400	Doramectin	-113.4
36294	DB00684	Tobramycin	-113.3
135564847	DB11401	Efrotomycin	-112.9
43708	DB00229	Cefotiam	-112.7

135398671	DB06827	Viomycin	-112.6
3002190	DB00976	Telithromycin	-112.4
127527	DB01329	Cefbuperazone	-112.3
6433412	DB11383	Carbomycin	-112.2
135431094	DB04934	Rifalazil	-112
43507	DB00923	Ceforanide	-111.9
135403821	DB01201	Rifapentine	-111.9
6436173	DB01220	Rifaximin	-111.8
65947	DB11892	Prulifloxacin	-111.4
19150	DB00689	Cephaloglycin	-111.2
47499	DB04570	Latamoxef	-111.2
53234134	DB09050	Ceftolozane	-111.1
54675777	DB09093	Chlortetracycline	-111.1
54680690	DB00618	Demeclocycline	-110.8
71141	DB09062	Cefminox	-110.7
11224409	DB12339	Radezolid	-110
9571074	DB01066	Cefditoren	-109.7
8378	DB00994	Neomycin	-108.8
15574941	DB08795	Azidocillin	-108.6
77843966	DB14879	Cefiderocol	-108.2
33255	DB01327	Cefazolin	-108.1
49800004	DB00781	Polymyxin B	-108.1
37768	DB00479	Amikacin	-107.6
441139	DB14057	Valinomycin	-107.6
43594	DB01328	Cefonicid	-107.4
39031	DB13693	Sulbenicillin	-107.4
54681908	DB12035	Sarecycline	-107
24847675	DBCAT001413	Virginiamycin	-106.7
150610	DB00303	Ertapenem	-106.5
53025	DB01330	Cefotetan	-106.4
71674	DB11375	Avilamycin	-106.1
11476460	DB09042	Tedizolid phosphate	-105.9
5284530	DB13499	Cefsulodin	-105.6
44187	DB01329	Cefoperazone	-105.3
54726192	DB12329	Eravacycline	-105.3
11979535	DB13704	Pristinamycin	-105.1
5388937	DB01369	Quinupristin	-104.8
6032	DB01172	Kanamycin	-104.7
135509117	DB04124	Aurodox	-104.5
33672	DB13506	Carfecillin	-104.4
5362065	DB00671	Cefixime	-104.4
456255	DB01326	Cefamandole	-104.2

3037209	DB13540	Isepamicin	-104.2
135413542	DB04918	Ceftobiprole	-103.9
20054942	DB11520	Hygromycin B	-103.8
446495	DB02237	Maltotetraose	-103.7
135413543	DB14733	Ceftobiprole medocaril	-103.5
3197	DB15582	Echinomycin	-103.5
6436055	DB13461	Cefcapene	-103.4
636405	DB00430	Cefpiramide	-103.1
71447	DB13814	Talampicillin	-102.5
33478	DB01604	Pivampicillin	-102.1
5459319	DB01669	Virginiamycin M1	-101.9
165580	DB01421	Paromomycin	-101.8
54680675	DB00453	Clomocycline	-101.7
461399	DB13261	Sitafloxacin	-101.5
135564528	DB15213	25-desacetylrifapentine	-101.4
444022	DB12127	Sultamicillin	-101.3
60651	DB11426	Marbofloxacin	-100.9
5479539	DB13682	Cefpirome	-100.8
5282165	DB01321	Josamycin	-100.6
6442177	DB01590	Everolimus	-100.3
104838	DB01598	Imipenem	-100
135398743	DB00615	Rifabutin	-100
487101	DB11943	Delafloxacin	-99.9
439318	DB13673	Bekanamycin	-99.8
8378	DB00452	Framycetin	-99.6
23665731	DB14725	Cefamandole nafate	-99.5
11993740	DB06600	Nemonoxacin	-99.4
6441094	DB11554	Tylvalosin	-99.3
68682	DB06696	Arbekacin	-99.2
6918267	DB06587	Mitemcinal	-99.2
9832301	DB11474	Tulathromycin A	-99.2
5379	DB01044	Gatifloxacin	-99.1
6321424	DB00602	Ivermectin	-99
54675783	DB01017	Minocycline	-99
6196	DB00713	Oxacillin	-99
54675779	DB00595	Oxytetracycline	-99
58258	DB13772	Rufloxacin	-98.9
60021	DB01405	Temafloxacin	-98.9
54708748	DB04785	Streptolydigin	-98.8
11234049	DB14569	Tedizolid	-98.8
54675776	DB00759	Tetracycline	-98.7
60605	DB11443	Orbifloxacin	-98.6

54675769	DB01051	Novobiocin	-98.4
72495	DB06461	Squalamine	-98.4
4628	DB13627	Oxolinic acid	-98.2
5284616	DB00877	Sirolimus	-98.1
9571107	DB01155	Gemifloxacin	-98
2764	DB00537	Ciprofloxacin	-97.8
65864	DB11935	Flomoxef	-97.4
21319	DB00301	Flucloxacillin	-97.4
152946	DB00218	Moxifloxacin	-97.4
5904	DB01053	Benzylpenicillin	-97.2
6869	DB00417	Phenoxymethylpenicillin	-97.2
6410758	DB13266	Cefatrizine	-97.1
38103	DB01333	Cefradine	-97.1
5481173	DB00438	Ceftazidime	-97
3085092	DB11544	Salinomycin	-97
11158972	DB15527	Umirolimus	-97
71339	DB13028	Biapenem	-96.9
4410	DB12447	Nadifloxacin	-96.7
34230	DB14056	Nigericin	-96.7
25242512	DB09308	Solithromycin	-96.5
6249	DB00415	Ampicillin	-96.3
71736	DB13778	Cefazedone	-96.3
5361871	DB13470	Cefodizime	-96.3
656580	DB07565	Chloramphenicol succinate	-96.2
71309	DB11485	Ceftiofur	-96
656511	DB00948	Mezlocillin	-96
36119	DB12604	Sisomicin	-96
3000226	DB02703	Fusidic acid	-95.8
42008	DB00274	Cefmetazole	-95.7
5282045	DB06419	Cethromycin	-95.7
5280757	DB11752	Bryostatin 1	-95.6
91810695	DBCAT000016	Mirvetuximab Soravtansine	-95.6
439542	DB00626	Bacitracin	-95.5
5284598	DBCAT001350	Oleandomycin	-95.5
9802884	DB11453	Pradofloxacin	-95.5
121904	DB04070	6-Deoxyerythronolide B	-95.4
9570757	DB00267	Cefmenoxime	-95.4
5281006	DB01150	Cefprozil	-95.3
6098	DB01147	Cloxacillin	-95.3
3229	DB00467	Enoxacin	-95.3
51081	DB00487	Pefloxacin	-95.2
3467	DB00798	Gentamicin	-95.1

6509979	DB00337	Pimecrolimus	-95.1
19649	DB01082	Streptomycin	-95.1
72396	DB04729	Gentamicin C1a	-95
6473883	DB00954	Dirithromycin	-94.7
171758	DB12343	Temocillin	-94.6
10034073	DB08874	Fidaxomicin	-94.5
11520894	DB06233	Ridaforolimus	-94.5
5388906	DB08903	Bedaquiline	-94.4
54682938	DB01301	Rolitetracycline	-94.4
60464	DB01208	Sparfloxacin	-94.3
5284619	DB06145	Spiramycin	-94.3
62959	DB00685	Trovafloxacin	-94.3
65452	DB11432	Narasin	-94.2
124093	DB06160	Garenoxacin	-94.1
445643	DB00864	Tacrolimus	-94
10178705	DB06771	Besifloxacin	-93.9
5282169	DB13456	Midecamycin	-93.8
33613	DB01060	Amoxicillin	-93.6
4583	DB01165	Ofloxacin	-93.1
5282521	DB11471	Tilmicosin	-93.1
6087	DB01603	Meticillin	-93
6533629	DB01332	Ceftizoxime	-92.8
93184	DB09319	Carindacillin	-92.7
470999	DB13270	Dibekacin	-92.7
5284585	DB00447	Loracarbef	-92.6
9578507	DB11459	Selamectin	-92.2
20824	DB00578	Carbenicillin	-92.1
8982	DB00607	Nafcillin	-92.1
287180	DB00817	Rosoxacin	-92.1
62858	DB13294	Azidamfenicol	-91.9
3037206	DB13274	Micronomicin	-91.9
446541	DB01024	Mycophenolic acid	-91.9
54697325	DB12455	Omadacycline	-91.6
5742832	DB00355	Aztreonam	-91.5
5742673	DB00493	Cefotaxime	-91.4
6918462	DB01256	Retapamulin	-91.4
6024	DB00456	Cefalotin	-91
6918289	DB06287	Temsirolimus	-90.8
6604200	DB00698	Nitrofurantoin	-90.4
5479537	DB01413	Cefepime	-90.3
71961	DB13816	Aspoxicillin	-90.2
47965	DB01140	Cefadroxil	-90

439530	DB08437	Puromycin	-90
3374	DB08972	Flumequine	-89.9
11567473	DB09047	Finafloxacin	-89.8
42613186	DB12615	Plazomicin	-89.8
4831	DB13823	Pipemidic acid	-89.7
52918384	DB11912	Lanopepden	-89.6
5360807	DB11423	Lasalocid	-89.5
44205191	DB13077	LCB01-0371	-89.2
6475694	DB13287	Miocamycin	-89.2
5281078	DB00688	Mycophenolate mofetil	-89.1
123865	DB04263	Geneticin	-89
45266800	DB00895	Benzylpenicilloyl polylysine	-88.6
6335986	DB01416	Cefpodoxime	-88.4
60063	DB14025	Clinafloxacin	-88.4
54676539	DB13092	Meclocycline	-88.3
5326	DB06821	Sulfameter	-88.1
36273	DB01163	Amdinocillin	-88
441306	DB00955	Netilmicin	-88
441199	DB01331	Cefoxitin	-87.7
2762	DB00827	Cinoxacin	-87.7
6918835	DB05903	KOS-1584	-87.6
19003	DB01000	Cyclacillin	-87.4
4539	DB01059	Norfloxacin	-87
71392	DB13300	Epicillin	-86.9
3084092	DB08965	Fusafungine	-86.9
71807	DB01147	Clometocillin	-86.8
11284169	DB12391	Sagopilone	-86.8
446596	DB00410	Mupirocin	-86.7
2794	DB00845	Clofazimine	-86.6
4421	DB00779	Nalidixic acid	-86.6
5486821	DB13504	Cefetamet	-86.5
5479529	DB01112	Cefuroxime	-86.5
72392	DB04808	Neamine	-86.4
91562	DB01414	Cefacetrile	-86.1
30699	DB01139	Cefapirin	-86.1
10250769	DB13739	Penamecillin	-86.1
2955	DB00250	Dapsone	-86
443387	DB00739	Hetacillin	-85.9
448013	DB03010	Patupilone	-85.9
43672	DB00319	Piperacillin	-85.8
3000540	DB01627	Lincomycin	-85.6
1046	DB00339	Pyrazinamide	-85

27447	DB00567	Cephalexin	-84.9
65957	DB11774	Pazufloxacin	-84.9
149096	DB01137	Levofloxacin	-84.8
5325	DB01581	Sulfamerazine	-84.6
71188	DB11404	Enrofloxacin	-84.4
56208	DB11491	Sarafloxacin	-84.3
33042	DB03615	Ribostamycin	-84
5284529	DB11367	Cefroxadine	-83.6
5215	DB00359	Sulfadiazine	-83.6
68595	DB11525	Maduramicin	-83.4
5959	DB00446	Chloramphenicol	-83.3
115163	DB01605	Pivmecillinam	-83.3
9568512	DB12829	Amithiozone	-82.9
3948	DB00978	Lomefloxacin	-82.8
5773	DB09008	Cefaloridine	-82.7
59364992	DB11416	Gamithromycin	-82.5
441401	DB00601	Linezolid	-82.5
24860548	DB11470	Tildipirosin	-82.5
6713928	DB13836	Metampicillin	-82.2
6662	DB14033	Acetyl sulfisoxazole	-82.1
3998	DB06795	Mafenide	-82
92879	DB13660	Propicillin	-82
5320	DB00634	Sulfacetamide	-82
447043	DB00207	Azithromycin	-81.9
5903	DB09320	Procaine benzylpenicillin	-81.9
73303	DB06211	Doripenem	-81.5
272833	DB13337	Pheneticillin	-81.5
18381	DB00485	Dicloxacillin	-81.4
441130	DB00760	Meropenem	-81.4
4855	DB13744	Piromidic acid	-81.4
56206	DB11511	Difloxacin	-81.2
447865	DB01873	Epothilone D	-81
253602	DB07374	Anisomycin	-80.8
51039	DB00833	Cefaclor	-80.5
3357	DB04576	Fleroxacin	-80.4
36921	DB01607	Ticarcillin	-80.1
9837656	DB05814	GPI-1485	-79.9
6445540	DB04845	Ixabepilone	-79.9
3767	DB00951	Isoniazid	-79.4
5426	DB01041	Thalidomide	-79.3
446598	DB01190	Clindamycin	-79.2
8359	DB11593	Isatoic anhydride	-79.2

46836890	DB11744	Epetraborole	-78.8
72474	DB00365	Grepafloxacin	-78.4
15541	DB00919	Spectinomycin	-78.1
114811	DB11413	Florfenicol	-77.7
157385	DB11537	Pirlimycin	-77.6
5282211	DB13409	Rokitamycin	-77.6
5344	DB00263	Sulfisoxazole	-77.5
441244	DB05245	Silver sulfadiazine	-77.4
5479	DB00911	Tinidazole	-77.2
5329	DB01015	Sulfamethoxazole	-76.6
5330	DB13773	Sulfamethoxypyridazine	-75.8
4649	DB00233	Aminosalicylic acid	-75.2
5104	DB11458	Roxarsone	-75.1
162290	DB13705	Decamethoxine	-75
72172	DB03424	Ubenimex	-75
5333	DB00259	Sulfanilamide	-73.9
5287620	DB07348	Brefeldin A	-73.7
443604	DB11468	Tiamulin	-72.9
572	DB02948	Fosmidomycin	-72.7
4173	DB00916	Metronidazole	-72.2
5280980	DB00766	Clavulanic acid	-71.9
27200	DB08621	Thiamphenicol	-70.8
6234	DB00260	Cycloserine	-68.5
14052	DB00330	Ethambutol	-68.4
4101	DB06799	Methenamine	-68
2722	DB01243	Chloroxine	-64.1
666418	DB12667	Protionamide	-63.5
446987	DB00828	Fosfomycin	-62.3
2761171	DB00609	Ethionamide	-58.3
176	DB03166	Acetic acid	-42.3
12560	DB00199	Erythromycin	14.3
202225	DB13179	Troleandomycin	16.2
444037	DB00778	Roxithromycin	89.4
71260	DB13338	Flurithromycin	146
84029	DB01211	Clarithromycin	177.6
